# A behavioural activation intervention to increase engagement with life and wellbeing in older adults: Study protocol for a randomised controlled trial

**DOI:** 10.1186/s40359-022-00988-1

**Published:** 2022-12-05

**Authors:** Julia E. T. Scott, Trevor G. Mazzucchelli, Mary A. Luszcz, Ruth Walker, Tim D. Windsor

**Affiliations:** 1 College of Education, Psychology and Social Work, Flinders University, Adelaide, SA Australia; 2 School of Population Health, Curtin University, Perth, WA Australia; 3 Caring Futures Institute, College of Nursing and Health Sciences, Flinders University, Adelaide, SA Australia

**Keywords:** Protocol, RCT, Older adults, Activity engagement, Wellbeing, Behavioural activation, Positive psychology

## Abstract

**Background:**

Meaningful activity engagement is a critical element of ageing well. Interventions designed to increase activity engagement tend to be activity-specific and do not always meet the needs of older adults with diverse interests and capacities. Behavioural activation (BA) provides a promising person-centred framework for promoting engagement in valued activities. This study will examine the effectiveness of a behavioural activation-based intervention for promoting engagement with life and wellbeing among older adults.

**Method:**

Participants will be randomly allocated to one of two conditions (BA intervention, Active Control) and take part in a six-week intervention that consists of one-on-one weekly sessions of up to one hour to be administered either via telephone or online video conferencing with a trained facilitator. This study will recruit 120 + participants aged 65 + who score at or below the median on a test of life engagement. Participants will complete questionnaires of primary and secondary measures both pre-program, one-week and three months post-program. Participants will also complete a daily diary questionnaire during the fourth and fifth weeks of the intervention. The primary outcome measure is the Life Engagement Test, and secondary outcome measures include assessments of subjective wellbeing, psychological wellbeing, mental health, self-reported health, social engagement, loneliness and life satisfaction.

**Discussion:**

The outcomes from this study will provide evidence as to whether a BA based approach represents an effective method for promoting engagement with life and wellbeing among older community-dwelling adults.

***Trial registration*:**

Australian New Zealand Clinical Trials Registry (Reg no. ACTRN 12621001192875). Trial retrospectively registered 6th September, 2021.

## Background

One factor that frequently features in definitions of what it means to age well is maintaining active engagement with life [1, 2]. Engagement with life refers to participating in multiple forms of valued activity, including social and solitary activity, productive engagement (e.g., paid work or volunteering), and/or contributing to the wellbeing of others and the community [1]. Ongoing engagement is believed to have a positive effect on physical health [3], cognitive function[4, 5] and psychological wellbeing [6]. Older adults themselves have also regularly identified active engagement with life as central to ageing well [7, 8].

Despite the accepted importance of remaining engaged for ageing well, changes and transitions that commonly occur in older adulthood can sometimes hinder engagement in meaningful activity. For example, losses in physical health and functional capacities can have a direct or indirect impact on activity engagement (e.g., through negative effect on access, or changes to caring responsibilities [9, 10]). Other common transitions that occur in older adulthood such as retirement, bereavement, or downsizing may affect activity engagement through their impact on social networks or finances [11, 12]. Additionally changes to society, such as lockdowns triggered by the COVID19 pandemic, can disproportionately impact older adults, who may be subject to stricter lockdown requirements, less likely to qualify for essential work exemptions, and may be less familiar with online alternatives to staying connected with friends and family [13]. The extent to which ageing presents challenges for ongoing engagement with life is reflected in research on social isolation (i.e., a lack of engagement with social networks). For example, social isolation was reported to increase from 5.4% among 18–39 year-olds to 21.7% in 70–79 year-olds in a recent population-based study of older Germans [14]. A recent US study showed similar rates of isolation among adults aged 65 and older, with almost one-in-four (24%) characterised as socially isolated [15].

Despite age-related challenges, ongoing activity engagement represents an element of ageing well that may be highly amenable to intervention. Multiple programs have been trialled, but these tend to be activity specific (e.g., [16–18]) and as such are not universally appropriate for people with different preferences or abilities. Furthermore, activity-specific programs that follow a “one-size-fits-all” approach may be less likely to fulfill psychological needs of competence, relatedness and autonomy that are important for autonomous motivation, whereby activity is volitional and has intrinsic value [19].

### Behavioural activation as a person-centred meaningful activity intervention

To address this need for a person-centred activity engagement intervention among older adults we have designed an intervention based on the principles of behavioural activation (BA). BA therapy was originally designed to treat depression, and can be used successfully alone, or in conjunction with other cognitive behavioural strategies [20, 21]. The BA model assumes that depression is maintained through a decrease in reinforcing activity and an increase in avoidance behaviours that often occurs with depressive symptoms. Low positive reinforcement increases the symptoms of depression, which can cause the person to withdraw from reinforcing behaviour even further, creating a negative feedback loop. BA assists clients to interrupt that negative cycle by proactively scheduling pleasurable and meaningful activities to support a lifestyle that is more reinforcing, which can reduce symptoms of depression and increase capacity for more reinforcing activity. BA includes activity monitoring to help participants identify patterns in their behaviours, to observe where and how their schedules could incorporate more pleasure or meaning, and to note the connections between activity engagement and mood. BA also includes personal values exploration to identify activities that will be the most reinforcing to an individual and incorporates problem solving to overcome barriers to activity engagement such low motivation, poor energy and concentration levels [22]. Importantly, given the focus of the present study, BA has also been found to be effective among community samples where the focus of treatment is shifted from treating depression to promoting wellbeing [20, 23]. Among older adults, BA has been successfully used to improve quality of life, functional disability and cognitive performance [24, 25]. Whether BA-based approaches provide a direct means of enhancing engagement with life more generally among older community-dwelling adults has not yet been examined.

We propose that the BA approach can have utility not just in reducing depressive symptoms, but also in supporting older adults’ engagement with life (and in turn wellbeing) by facilitating processes of effective self-regulation. A central means through which older adults adapt to changing life circumstances is through regulating their goals in ways that provide an optimal fit with their available resources [26, 27]. For example, successful development depends on making sound judgements about when to invest resources such as time, energy and money into goals that are valued and achievable (assimilation), and when to abandon and de-value goals that are no longer attainable (accommodation, [28]). Importantly, when goals are abandoned, successful development often depends on the ability to reengage with new goals [29]. For example, a person might mobilise their resources to support their efforts as part of an outdoor environmental volunteering group, but when physical limitations make the work no longer possible or enjoyable, this goal might be abandoned in favour of contributing to this (or another) cause by taking on an administration-focused role that requires less physical exertion.

The BA approach focuses on working with participants to identify their values, and activities that are consistent with those values (e.g., [20]). Participants are encouraged to reflect on the degree to which their current activities align with their values, and to identify and schedule activities that are important to them. The BA approach also involves identifying challenges to activities (i.e., blocked goals) and encourages participants to use effective strategies around selecting alternative values-consistent activities and identifying the supports (e.g., social support) needed to make them attainable. By supporting processes of self-regulation (assimilation, accommodation, goal re-engagement) in relation to activities that are values-consistent, we expect participation in a BA program to result in improvements in engagement with life among older adults.

### Study aims

The proposed randomised controlled trial has several objectives, specifically:

1. To determine the impact of a 6-week BA intervention compared to an active control wellbeing intervention on meaningful activity engagement among older adults from the general population at both one week and three months post-intervention.
2. Determine the impact of BA compared to active control on secondary measures of wellbeing, psychosocial functioning and health, including subjective wellbeing (positive and negative affect and life satisfaction), psychological well-being (flourishing, self-compassion) social engagement and loneliness, mental health (symptoms of depression and anxiety), aspects of self-regulation (goal disengagement and goal re-engagement) and self-reported health.
3. Gain further feedback about program acceptability through a participant survey at the completion of the program.

It is hypothesised that the BA intervention will have a greater positive impact on meaningful activity engagement compared to the active control condition at one week follow-up. This was based on the brief positive psychology interventions in the active control condition being selected on the basis that they aim to increase wellbeing, but do not directly target engagement with activities to enhancing wellbeing.

Participants will also complete a 14-day daily questionnaire (see methods) focusing on daily exposure to stressful events, self-compassion and affect.

Finally, researchers have emphasised the value in conducting intensive longitudinal research as part of clinical trials as a means of providing more ecologically valid data that can be used to examine within-person processes (e.g., [30]). Building on emerging research concerned with short-term variability in self-compassion (e.g., [31]) the study will also include a 14-day daily diary embedded within the larger project. As an observational sub-component of the larger trial, the daily diary data component will allow examination of short-term daily linkages between stressor exposure and affect, and whether affective reactivity to stressors is lower on days when participants report higher self-compassion. Although it will be necessary to control for treatment condition in these analyses, we do not have a priori expectations regarding possible treatment effects. These analyses will be exploratory and do not relate directly to evaluation of the intervention; details of the daily diary component are reported below for completeness.

## Methods/design

### Design

The ELMS is a randomised control trial, that investigates whether an intervention based on BA principles improves life engagement and wellbeing compared to an active control condition, specifically a multi-component positive psychology intervention (MCPPI). Participants will be randomly allocated to one of the two conditions (BA, MCPPI) and will take part in an intervention consisting of six weekly one-on-one sessions of up to one hour, to be administered by trained facilitators via telephone or web conferencing (based on participants’ preference). Program efficacy will be determined by assessment at three time points: baseline (pre-intervention), 1 week and 3 months post intervention. All participants will also complete a daily questionnaire for two weeks between sessions 4 and 6. A summary of the study periods and assessments are provided in Table 1.

**Table 1:** Summary of study phases and assessment measures

Time point	Pre-program	Baseline	Program (6 weeks)	1-week post-program	3-month post-program
Enrolment					
Eligibility screen (Life Engagement Test)^a^	X				
Informed consent	X				
Allocation to condition	X				
Intervention					
Treatment: BA			↔		
Active control: MCPPI			↔		
MOCA-blind			X (wk 5)		
Daily questionnaire (2 weeks)			X (wk 4–6)		
Assessment					
Demographics		X			
Alcohol and tobacco use		X			
Pain and physical health		X		X	X
SPANE		X		X	X
Self-compassion scale		X		X	X
BADS		X		X	X
HADS		X		X	X
Life engagement test		X		X	X
UCLA loneliness scale		X		X	X
Flourishing scale		X		X	X
Satisfaction with life scale		X		X	X
Engaged living scale		X		X	X
Social networks scale		X		X	X
Goal adjustment scale		X		X	X
Client satisfaction questionnaire				X	
Daily questionnaire assessment					
State self-compassion			X		
Daily affect scale			X		
DISE			X		
Eudaimonic wellbeing			X		

*BA* Behavioural activation;* BADS*  Behavioural Activation for Depression Sale;* DISE*  Daily Inventory of Stressful Events;* HADS* Hospital Anxiety and Depression Scale;* LET* Life Engagement Test;* MCPPI* Multi component positive psychology intervention;* SPANE* Scale of Positive and Negative Experience

^a^Participants are eligible if they score at or below the mean LET reported in previous studies (see *Participants*)

### Participants

Older adults aged 65 + from the general population, who have a purpose in life (Life Engagement Test) score at or below the population mean in previous community samples [32] will be eligible to participate. Those with substantial hearing loss, difficulty with English or advanced cognitive impairment (or any other difficulties that meant they could not feasibly take part in a telephone-based intervention, as determined by either the recruiter, or the facilitator at first contact) would not be eligible to participate. Potential participants will be initially identified utilising a list of older adults who have agreed to be contacted for future research participation, maintained by the Adult Development Lab at Flinders University, Adelaide, South Australia as well as through advertisements in Weekend Plus, a digital magazine for older adults produced by the South Australian Government, and email invitations sent to online networks such as the South Australian Office for Ageing Well Feedback Network (a group of older adults who have indicated a willingness to be contacted about research projects or government initiatives). Participants will either be contacted by telephone (e.g., those on our database) or will complete an online screening survey to determine eligibility. To date, all participants recruited have resided within South Australia in either metropolitan, regional and rural areas. Future participants may also be recruited through industry partner organisations such as ECH Inc.

### Behavioural activation intervention

The behavioural activation intervention was adapted from the Revised Behavioural Activation Treatment for Depression manual [33]. Detail about the development of the program through piloting and the adaptions made from the original manual have been provided elsewhere [34]. The primary change to the program was the refocus of content toward improving wellbeing and sense of purpose through meaningful activity engagement, as opposed to a focus on alleviating feelings of depression and low mood. Participants will be provided with a treatment manual to help guide their progress. Based on participant feedback from the pilot study, some minor changes were made to the manual, specifically some content and repetition was removed for brevity, and some language was updated to improve readability.

A breakdown of the six sessions of this intervention outlined in the participant treatment manual are provided in Table 2. Briefly, during the first session participants will be introduced to the concept of wellbeing, the behavioural activation approach, and the activity monitoring. During the second session they will be guided through values identification, and brainstorming activities consistent with their values. They also review their activity monitoring charts and continue with monitoring in the following week. During the third session, participants will begin to select and rank activities according to difficulty and feasibility. They are also encouraged to schedule some of these activities into their monitoring sheets. At the fourth session, participants will review their activities over the previous week, troubleshoot potential barriers and consider seeking support from others, if required. The fifth session consists of a review of values and activities and troubleshooting barriers. The final session will review progress made, and how to apply these skills in the future.

**Table 2:** Overview of behavioural activation intervention

Week	Content	Estimated duration
1. Wellbeing	What is wellbeing? The activation approach to wellbeing Stressful events and loss Learning your patterns of behaviour: Introduction to daily monitoring	60 min
2. Life areas, values, and activities	Review of monitoring Troubleshooting problems with monitoring Life areas, values, and activities	60 min
3. Activity selection and ranking	Review of monitoring Review of life areas, values, and activities inventory Activity selection and ranking Daily monitoring and activity planning	60 min
4. Reviewing achievements and seeking support from others	Review of monitoring / activities Troubleshooting problems with activities Seeking support from others Daily monitoring with activity planning	45 min
5. Reviewing achievements and modifying activities	Review of monitoring / activities Troubleshooting problems with activities Review of life areas, values, and activities Review of activity selection and ranking Daily monitoring with activity planning	15–30 min
6. Beyond the program	Review of monitoring / activities Troubleshooting problems with activities Daily monitoring with activity planning Review of progress Preparing for the future	15–30 min

### Active control (multi-component positive psychology intervention)

Inclusion of an active control condition that is comparable to the intervention in terms of the required time, effort, attention and amount of content means that any differences in outcomes between the groups can be attributed to the intervention content, as opposed to non-specific treatment effects [35]. An active control condition may also be more ethical than other forms of control condition as all participants will receive a meaningful intervention upon enrolment into the study [36, 37]. Lastly, as both the treatment and control conditions provide a meaningful wellbeing intervention, participants will be blinded to their allocation to the intervention or control condition, thereby reducing expectation confounds. For the active control in the current study a MCPPI was designed. Positive psychology is a therapeutic approach that aims to improve wellbeing and promote flourishing, as opposed to treating mental illness [38]. MCPPIs, which typically incorporate multiple short positive psychology interventions that aim to generate specific positive emotions, have been found to be effective in increasing wellbeing, especially compared to other therapeutic approaches [23].

The specific concepts and activities included within this intervention were selected according to: (a) their comparative effectiveness based on recent meta-analytic findings [39], (b) their emphasis on cognitive experiences and evaluations, as opposed to behaviour and activity, to minimise content overlap with the BA condition, and; (c) provide a variety of activities to increase the chance that participants will find at least one intervention that appeals to them, or that they find helpful [40]. Original sources for the activities that were incorporated can be found in Table 3. Activities listed in Table 3 without citations were developed or modified for the active control condition.

**Table 3:** Overview of multi-component positive psychology active control intervention

Week	Content	Estimated duration
1.Wellbeing, positive emotion and positive psychology	What is wellbeing? Introduction to positive psychology The benefits of positive emotion Reflection	60 min
2. Gratitude and optimism	Review of previous week Gratitude Activities to increase gratitude 3 Good things^1^ Expressing gratitude^2^ Optimism Optimism reflection	60 min
3. Character strengths and forgiveness	Review of week and activities Character strengths and identification Character strength task^3^ Forgiveness Forgiveness reflection	45 min
4. Savouring	Review of week and activities Introduction to savouring Savouring the past activity^4^ Savouring the present activity^5^ Savouring the future activity	45 min
5. Reviewing achievements, troubleshooting and modifying activities	Review of week and activities Troubleshooting problems with activities	15–20 min
6. Beyond the program	Review and reflection of activities Review of progress Preparing for the future	15–30 min

^1^ Seligman ME, Steen TA, Park N, Peterson C. Positive psychology progress: empirical validation of interventions. American psychologist. 2005;60(5):410

^2^Parks AC, Schueller S. The Wiley Blackwell handbook of positive psychological interventions: John Wiley & Sons; 2014.; Lyubomirsky S, Dickerhoof R, Boehm JK, Sheldon KM. Becoming happier takes both a will and a proper way: an experimental longitudinal intervention to boost well-being. Emotion. 2011;11(2):391

^3^Seligman ME, Rashid T, Parks AC. Positive psychotherapy. American Psychologist. 2006;61(8):774

^4^Palmer CA, Gentzler AL. Adults’ self-reported attachment influences their savoring ability. The Journal of Positive Psychology. 2018;13(3):290–300

^5^Smith JL, Hanni AA. Effects of a savoring intervention on resilience and well-being of older adults. Journal of Applied Gerontology. 2019;38(1):137−52

A summary of the six sessions contained in the MCPPI participant treatment manual is provided in Table 3. In the first session, participants will be introduced to the concept of wellbeing, positive psychology and the role of positive emotions. In Sessions 2 through 4, participants will be introduced to one or more types of positive emotions and how those emotions may be of benefit. Participants will then be then provided with a reflection exercise or an activity to help cultivate that emotion. In session five, participants will be encouraged to reflect on the activities learned and troubleshoot and modify activities as required. In the sixth session participants will review the progress made and consider how to apply these skills going forward. Due to resource constraints, participants in either condition will not be offered the opportunity to participate in the alternative condition.

### Assessment

Study outcomes will be assessed via surveys, completed online, or in paper-and-pencil format, depending on participants’ preferences. Initial efficacy of the behavioural activation intervention will be assessed at the 1-week post-program assessment. Data from the 3-month follow-up assessment will be used to examine longer-term durability of effects beyond the 1-week follow-up. Measures are detailed below.

#### Primary outcome measure

The Life Engagement Test (LET; [41]) is a self-report questionnaire measure that will be used to assess the extent to which a person perceives that the activities they engage in are personally valued and meaningful. This scale consists of six items (e.g., “I value my activities a lot”), rated on a 5-point Likert scale (1 = *strongly disagree*, to 5 = *strongly agree*). The LET is scored in two steps. First, items 1, 3, and 5 are reverse coded. Second, the six items are summed such that higher scores indicate a stronger perception that one’s activities are valued. The total scale score will be used. The LET has been demonstrated to have good internal consistency (average α = 0.80) and convergent and discriminant predictive validity [41].

#### Secondary outcome measures

Social engagement will be measured via a series of questions on network size, relationship quality, and contact frequency that were used within the English Longitudinal Study of Ageing [42], and the Health and Retirement Study [43]. The questions are provided three times, with reference to children, other immediate family and friends, respectively. Network size is assessed by two questions that assess the number of (children/immediate family/friends) with whom participants have contact, and a close relationship. Six questions explore both positive and negative qualities of these relationships (e.g., “How much do they really understand the way you feel about things?”) and answers are rated on a 4-point scale (1 = *a lot* to 4 = *not at all*). Contact frequency with children/immediate family/friends was assessed by three questions where participants indicate how frequently they (a) meet up, (b) speak on the phone, and (c) write or email.

Loneliness will be measured using the UCLA Loneliness Scale 10-item version (ULS-10; [44]) will be used to measure the subjective experience of loneliness. This scale consists of 10 items (e.g., “How often do you feel that no one really knows you well?”), which are rated on a 4-point scale (1 = *Never*, 2 = *Rarely*, 3 = *Sometimes*, 4 = *Always*). The 10-item short version of this measure shows good internal consistency (α = 0.89; [44]).

Subjective and psychological wellbeing will be assessed using a number of measures. Affect will be measured using the Scale of Positive and Negative Experience (SPANE; [45]), which includes six items that measure positive feelings (e.g., “pleasant”, “contented”) and six items that measure negative feelings (e.g. “sad”, “angry”). Participants rate how frequently they experienced each feeling over the past 4 weeks using a 5-point scale (1 = *very rarely or never* to 5 = *very often or always*). The scale is reported to have good internal consistency (α = 0.81 to 0.89; [45]) and convergent validity [46]. The Satisfaction with Life Scale [47] comprises five items (e.g., “I am satisfied with my life”) scored using a 7-point scale (1 = *strongly disagree* to *strongly agree*). Higher scores indicate greater life satisfaction (Diener et al. 1985). The scale has been demonstrated to have good internal consistency (α = 0.84; Steger et al., 2006) and correlates positively with optimism and self-esteem (Steger et al., 2006). The Flourishing Scale [45] includes five items (e.g., “I am a good person and live a good life”) and is scored using a 7-point scale (1 = *strongly disagree* to 7 = *strongly agree*). Higher scores indicate greater wellbeing. This scale has good internal consistency (α = 0.84) and convergence with other wellbeing scales [45]. The Self-Compassion Scale Short Form [48] is a 12-item measure that will be used to assess levels of trait self-compassion. Participants indicate how often they act in accordance with a series of statements (e.g., “When something painful happens I try to take a balanced view of the situation”) on a 5-point scale (1 = *almost never* to 5 = *almost always*). This scale has good internal consistency (α ≥ 0.86) and a near-perfect correlation with the long form self-compassion scale (*r* ≥ .97 all samples [48].

Physical health will be assessed via the Medical Outcomes Study Physical Functioning Subscale [49], which is a 10-item measure of physical activity limitations. Participants indicate whether their health limits them in various activities (e.g., “Lifting or carrying groceries”) and if so, the severity of each limitation on a 3-point scale (1 = *yes, limited a lot* to 3 = *no, not limited at all*). The scale has been demonstrated to have strong construct and discriminant validity [50]. A single item measure taken from the Medical Outcomes Study Bodily Pain Subscale was also used to measure pain. Participants indicate “How much bodily pain have you had in the last four weeks” on a 6-point scale (1 = *none* to 6 = *very severe*) [50]. Self-rated health was measured by a one-item measure “how would you rate your overall health at the present time”. Responses are rated on a 5-point scale (1 = *Poor* to 5 = *Excellent*).

Mental health will be assessed using the Hospital Anxiety and Depression Scale [51], which includes two 7-item subscales that measure symptoms of depression and anxiety (e.g., “I feel tense or wound up”). This scale has good internal consistency (α ≥ 0.75 [52]) and convergent validity [53], and is widely used among older adult due to its low reliance on physiological symptoms in the assessment of anxiety and depression [52].

Self-regulation will be assessed by the Goal Adjustment Scale [29], a 10-item measure developed to assess reaction to life goal adjustments. Four items measure participants’ goal disengagement capacities (e.g., “It’s easy for me to reduce my effort towards the goal”), and six items measured their goal reengagement capacities (e.g., “I start working on other new goals”). Reponses are measured on 5-point Likert-type scales (1 = *strongly disagree* to 5 = *strongly agree*). The disengagement and reengagement subscales have adequate (α = 0.75) and excellent (α = 0.91) internal consistency respectively [54].

#### Mechanisms

Possible mechanisms will be further assessed via the Behavioural Activation Depression Scale—Short Form (BADS-SF), and the Engaged Living Scale-Short Form (ELS-SF). The BADS-SF [55] is a 9-item self-report scale that measures activation (i.e., goal oriented, values-consistent behaviour) and avoidance over the past week. Items are rated on a 7-point scale (0 = *not at all* to 6 = *completely*), and higher scores represent greater activation. The BADS-SF has good internal consistency (α = 0.82) and construct validity [55]. The ELS-SF [56] is a 9-item measure that assesses participation in valued activities. It comprises two subscales: “Valued Living” and “Life Fulfilment”. Participants indicate their level of agreement to a series of statements (e.g., “I make choices based on my values, even if it is stressful”) on a 5-point scale (1 = *completely disagree* to 5 = *completely agree*). This scale is reported to have good internal consistency for the total scale (α = 0.88) and subscales (Valued Living α = 0.76; Life Fulfilment α = 0.89) and has demonstrated both convergent and discriminant validity [56].

#### Other measures

At baseline, a brief demographic survey will also be used to assess sex, age, ethnicity, education, current employment status and disposable income. Participants will also be asked questions about housing tenure, and cigarette and alcohol consumption. At week five of the intervention, participants will also complete the MOCA-Blind [57], which is a cognitive screener that can be administered over the telephone. Lastly, a 19-item client satisfaction questionnaire will be administered at the first post-program assessment. The items, adapted from the form used by Mazzucchelli, Rees, and Kane [58], assess participant satisfaction, and perceived usefulness of program intervention. These items are rated on a 7-point scale (1 = *no, definitely not* to 7 = *yes, definitely*), with the final question inviting narrative feedback.

#### Daily questionnaire assessment

State self-compassion will be measured using the six item State Self-Compassion Scale—Short Form [59]. Participants indicate their agreement on a 4-point scale (1 = *not at all* to 5 = *to a great extent*) with statements that reflect the tendency to respond self-compassionately (e.g., “I’m giving myself the caring and tenderness I need”). This scale is highly correlated with the long form of the questionnaire, which demonstrated adequate internal consistency (α = 0.72 to 0.82; [59])

Daily affect will be assessed using a measure of positive and negative affect adapted from Hülür et al. [60]. This 8-item scale contains four items that measure positive feelings (e.g., “relaxed”, “interested”) and four that measure negative feelings (e.g., “lonely”, “irritable”). Participants rate how much they experienced each feeling that day using a 5-point scale (0 = *no experience of the affect* to 5 = *very intense experience of the affect*). The scale has been reported to have acceptable internal consistency for both the positive affect subscale (α = 0.78) and the negative affect subscale (α = 0.67;[60]).

Exposure to stressful events will be measured using a short form of the Daily Inventory of Stressful Events [61]. This questionnaire consists of five questions (e.g., “Did anything stressful happen to you with regard to your personal health?”) to which participants respond “yes” or “no”.

Eudaimonic wellbeing will be measured using two short subscales of Meaning in Life, “presence” and “search” as used in Nezlek, Newman, and Thrash [62]. The Presence subscale consisted of two items: “How meaningful did you feel your life was today” and “How much did you feel your life had purpose today?” The search subscale consisted of two items: “How much were you searching for meaning in your life today?” and “How much were you looking to find your life’s purpose today?”

### Study timeline

Older adults who are on a pre-existing list maintained by the Adult Development Lab will be initially contacted by telephone by a research assistant. They will be provided with details of the study, and if they indicate they are interested in participating, they will be administered the LET over the phone to assess their eligibility to participate. Participants who are recruited by online advertisements will be invited to follow a link where they will be provided some information about the study and invited to complete the LET online to determine eligibility. Participants undertaking the full 6-item LET at telephone screening will be eligible to participate if they score at or below 25. Participants undertaking the online screening will be administered a 5-item version of the LET and will be eligible if they score at or below 21 (the mean of the 5-item scale in a community sample of older adults assessed as part of the broader project, N = 431). The final LET item “I have lots of reasons for living” will be excluded for online participants to reduce the possibility of negative feelings among some participants, given that the item would not appear within the context of either a larger survey, or with the presence of the telephone interviewer as an immediate source of support and/or information. The decision to exclude the final item from the online screener was also based on previous data indicating strong item-specific ceiling effects (M = 4.43 (SD = 0.86) on a scale of 1 to 5; [32]) suggesting limited discriminatory value. All eligible participants will be sent, by post or email, the study information sheet, consent form and baseline questionnaire (marked with a unique 3-digit identifier) to complete and return. Those who elect to receive the baseline by email will be sent a link to the baseline questionnaire hosted on the Qualtrics Online Survey Platform. Once the baseline questionnaire is completed participants are considered fully recruited into the study. Using simple randomisation via Microsoft Excel, participants are then allocated to treatment condition. Randomly allocated to treatment condition, sent the corresponding intervention manual and allocated to a study facilitator. The facilitator will contact the participant by telephone to set up a time for the first session and will be responsible for all contact from that participant from then on.

**Fig. 1:**
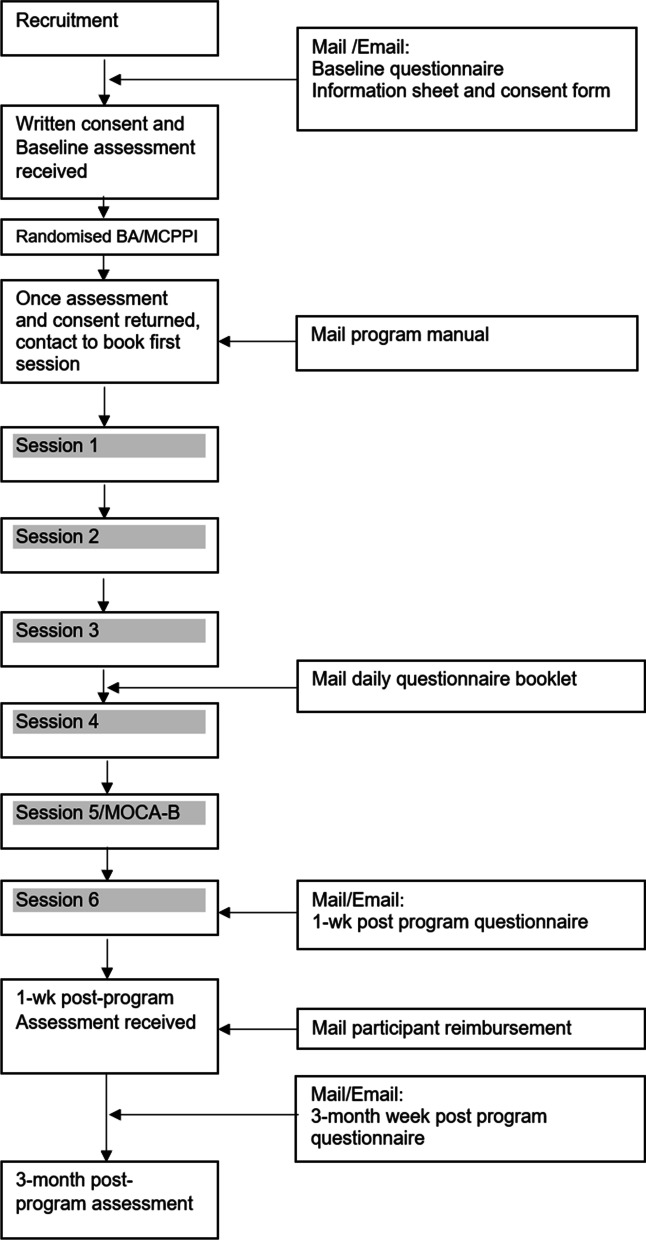
Study Timeline

Participants will then undergo six weekly sessions conducted either via the telephone or via online video conferencing software. A graphical representation of the study timeline is provided in Fig. 1. Between each session they will be provided with some activities to complete. For those in the BA group, the between session activities will be flagged as particularly important to complete. If they are not completed during the week, they will be completed with the facilitator during the next session, as per BA protocol [22]. The MCPPI group will be encouraged to complete the between session activities. Facilitators will track their progress on running sheets which will be used to guide content delivery, document participant adherence and make note of anything relevant that may impact delivery or progress. For two weeks during the fourth and fifth weeks of the intervention, participants will be asked to complete a daily questionnaire, which will be posted to them with a reply-paid envelope to return once completed. It is anticipated these questionnaires will take less than 5 min per day to complete. At the end of the fifth session, participants will undergo the MOCA-Blind [57]. Once they have completed the intervention, participants will be posted or emailed two follow-up questionnaires; the first at one week post intervention, and second at three months post intervention. It is anticipated these will take approximately 30 min each to complete. Once their first follow-up assessment is returned, participants will be sent $100 (AUD) reimbursement for their time in the form of a pre-loaded EFTPOS card.

Participants will be recruited in waves over the study period depending on the capacity of available facilitators. An initial “soft launch” of the program was conducted with eight participants with sessions facilitated by JETS (registered clinical psychologist). Some minor changes to text (for clarity) were made to the BA intervention manual after the soft launch. Facilitators will have at least honours-level qualifications in psychology, or undergraduate psychology with previous experience working with diverse clients (e.g., through volunteer telephone counselling) as well as online training in administration of the MOCA-Blind. Facilitators will receive project-specific training in principles underlying BA, the application of BA in the context of promoting wellbeing (as opposed to treating depression), positive psychology interventions and the structure and administration of the sessions in both the treatment, and active control conditions. Training will take place in small groups over half-a-day, led by JETS. To reduce potential biases in administration across different facilitators, and to promote treatment fidelity, facilitators will complete a running sheet that includes a checklist of key content for each session across the BA and MCPPI conditions (running sheets are available upon request to the corresponding author).

### Good clinical practice

Informed consent will be obtained from all participants once the study procedures, risks and their rights have been explained to them. Additionally, participants will be informed of the limits to confidentiality. If they reveal information that suggests there is a risk of harm to themselves, or others, facilitators will be required to inform emergency services. In addition, facilitators will also be required to report suspected cases of child abuse or neglect to the child abuse report line. Ongoing supervision for facilitators will be provided by JETS. This study has been approved by the Flinders University Social and Behavioural Research Ethics Committee (Study no. 8172). This trial has also been retrospectively registered with the Australian New Zealand Clinical Trials Registry (ACTRN12621001192875), as was the pilot study (ACTRN12620000126910).

### Sample size and analysis

Sample size considerations were based on the numbers of participants needed to detect a medium effect size difference between the BA and active control groups on the LET at 1-week post intervention. This represents a difference of approximately 2-units on the LET based on previous research (possible scores range from 6 to 30) [32]. Positive psychology interventions have typically been found to have effect sizes in the small to medium range; for example, a recent meta-analysis reported values ranging from *g* = 0.39 to *g* = 0.62 across mental health and well-being outcomes [39]. Within this range, we deemed a medium effect as being a reasonable marker of meaningful change in life engagement, given that our exclusion criterion (see *Participants*) excluded those already scoring at or near the maximum on the LET. Using the Stata “power” module with alpha = 0.05 and a group difference of *d* = 0.52 indicated that 60 participants per group are required for power = 0.8. Data will be analysed using linear mixed models, which allow for dependencies among repeated observations, as is the case with pre- and post-test data (e.g., [63]). Models will be specified taking into account guidelines provided by Twisk et al. [64] for estimating treatment effects in randomised controlled trials. Specifically, a mixed model will be specified that includes dummy variables for time (contrasting baseline with 1-week and 3-month follow-ups) and interactions of time with treatment (BA vs. MCPPI) to estimate treatment effects at the first (1-week) and second (3-month) follow-ups. This approach controls for treatment group differences at baseline and makes use of all available data, with missing data accommodated under FIML assumptions [65].

## Discussion

Despite the importance of meaningful activity engagement in older adulthood, there are limited interventions available that are person-centred rather than activity specific. Interventions that can be tailored to the unique interests, values and capacities of individuals may represent a promising approach to enhancing older adults’ engagement with life. This study will test the efficacy of BA as a person-centred approach to promoting engagement with life in older adulthood. We see the promise of the BA approach as primarily arising from (a) the fact that it can be tailored to the unique circumstances of individuals, (b) its delivery not requiring a high degree of clinical expertise among facilitators ([66]), and (c) its principles of administration mapping onto processes of self-regulation that are recognised as supporting effective development across the life course [26, 28]. BA has been found to be effective in improving wellbeing in non-clinical samples [20, 23], but there is scarce research to date on whether BA-based approaches have utility in promoting engagement among community-dwelling older adults. BA will be compared to an MCPPI which will act as an active control condition, which makes use of evidence-based approaches to facilitating well-being [39, 67]. In addition to directly assessing the effectiveness of BA on promoting engagement with life, the study includes several novel secondary outcome measures (e.g., flourishing, goal disengagement and re-engagement, self-compassion) that will allow for exploration of whether the BA approach offers benefits to well-being above and beyond any benefits provided by the MCPPI.

## Data Availability

Once collected, deidentified data will be available from the corresponding author on reasonable request.

## Abbreviations

BA: Behavioural activation
BADS-SF: Behavioural Activation Depression Scale – Short Form
ELMS: Engagement, Life and Meaning Study
ELS-SF: Engaged Living Scale – Short Form
LET: Life Engagement Test
MCPPI: Multi-component positive psychology intervention
MOCA-Blind: Montreal Cognitive Assessment - Blind
UCLA Loneliness Scale: University of California Los Angeles Loneliness Scale
